# Metabolically healthy and unhealthy obesity and the development of lung dysfunction

**DOI:** 10.1038/s41598-023-31960-7

**Published:** 2023-03-27

**Authors:** Jae-Uk Song, Jonghoo Lee, Si-Young Lim, Hyun-Il Gil, Yoosoo Chang, Seungho Ryu

**Affiliations:** 1 Division of Pulmonary and Critical Care Medicine, Department of Internal Medicine, Kangbuk Samsung Hospital, Sungkyunkwan University School of Medicine, 29 Saemunan-ro, Jongno-gu, Seoul, 03181 Republic of Korea; 2 Department of Internal Medicine, Jeju National University Hospital, Jeju National University School of Medicine, Jeju, Republic of Korea; 3 Center for Cohort Studies, Total Healthcare Center, Kangbuk Samsung Hospital, Sungkyunkwan University School of Medicine, 29 Saemunan-ro, Jongno-gu, Seoul, 03181 Republic of Korea; 4 Department of Occupational and Environmental Medicine, Kangbuk Samsung Hospital, Sungkyunkwan University School of Medicine, Samsung Main Building B2, 250, Taepyung-ro 2ga, Jung-gu, Seoul, 04514 South Korea; 5 Department of Clinical Research Design and Evaluation, SAIHST, Sungkyunkwan University, Seoul, Republic of Korea

**Keywords:** Respiratory tract diseases, Metabolic syndrome, Obesity

## Abstract

We investigated the association of metabolically healthy (MH) and unhealthy (MU) obesity with incident lung dysfunction. This cohort study included 253,698 Korean lung disease-free adults (mean age, 37.4 years) at baseline. Spirometry-defined lung dysfunction was classified as a restrictive pattern (RP) or obstructive pattern (OP). We defined obesity as BMI ≥ 25 kg/m^2^ and MH as the absence of any metabolic syndrome components with a homeostasis model assessment of insulin resistance < 2.5: otherwise, participants were considered MU. During a median follow-up of 4.9 years, 10,775 RP cases and 7140 OP cases develped. Both MH and MU obesity showed a positive association with incident RP, with a stronger association in the MU than in the MH group (*P*_interaction_ = 0.001). Multivariable-adjusted hazard ratios (95% CI) for incident RP comparing obesity to the normal-weight category was 1.15 (1.05–1.25) among the MH group and 1.38 (1.30–1.47) among MU group. Conversely, obesity was inversely associated with OP because of a greater decline in forced vital capacity than forced expiratory volume in 1 s. Both MH and MU obesity were positively associated with RP. However, the associations between obesity, metabolic health, and lung functions might vary depending on the type of lung disease.

## Introduction

Lung function impairment, in both obstructive and restrictive patterns, is associated with chronic respiratory and non-respiratory disease development, including deaths from all causes and cardiovascular disease^1–4^, contributing to significant public health problems worldwide^5,6^. Furthermore, chronic obstructive lung disease commonly manifests after the age of 40; however, there is growing attention that lung dysfunction occurs much earlier than overt disease manifestation^7^. Early identifying modifiable risk factors for lung function impairment and understanding its pathophysiology is important to establish preventive measures to reduce chronic respiratory disease and other non-respiratory complications.

Obesity and metabolic syndrome are associated with respiratory symptoms, lung disease, and spirometry lung function. Most studies have evaluated the effects of either obesity or metabolic syndrome on lung function separately with mixed results^1,6,8–14^. Obesity is often accompanied by metabolic abnormalities, such as hypertension, type 2 diabetes, insulin resistance, and dyslipidaemia. However, a subset of obese individuals do not always present with metabolic abnormalities despite having excessive body fat; this is referred to as metabolically healthy obesity (MHO), possibly contributing to favourable prognosis without adverse obesity-related outcomes^15^. Combined or isolated obesity phenotypes and metabolic health status may help elucidate whether obesity per se or the presence of co-existing metabolic abnormalities affects lung function impairment. However, to date, the longitudinal association between different metabolic health and obesity phenotypes and lung function impairment is generally unknown.

We investigated the longitudinal relationship between body mass index (BMI), a proxy indicator of obesity, and metabolic health status with different lung function impairment types in a large cohort of apparently healthy young and middle-aged Korean adults, lung-disease free at baseline, who participated in a comprehensive screening examination, including repeated spirometry measures.

## Methods

### Study population

The Kangbuk Samsung Health Study is a cohort study involving Korean men and women who underwent comprehensive health examinations at the Total Healthcare Center of Kangbuk Samsung Hospital clinics in Seoul and Suwon, South Korea since January 1, 2002^16^. More than 80% of the participants were employees of various companies, local governmental organisations, or their spouses. In Korea, annual or biennial employee health screenings are required by the Industrial Safety and Health Law and are provided free of charge.

**Figure 1:**
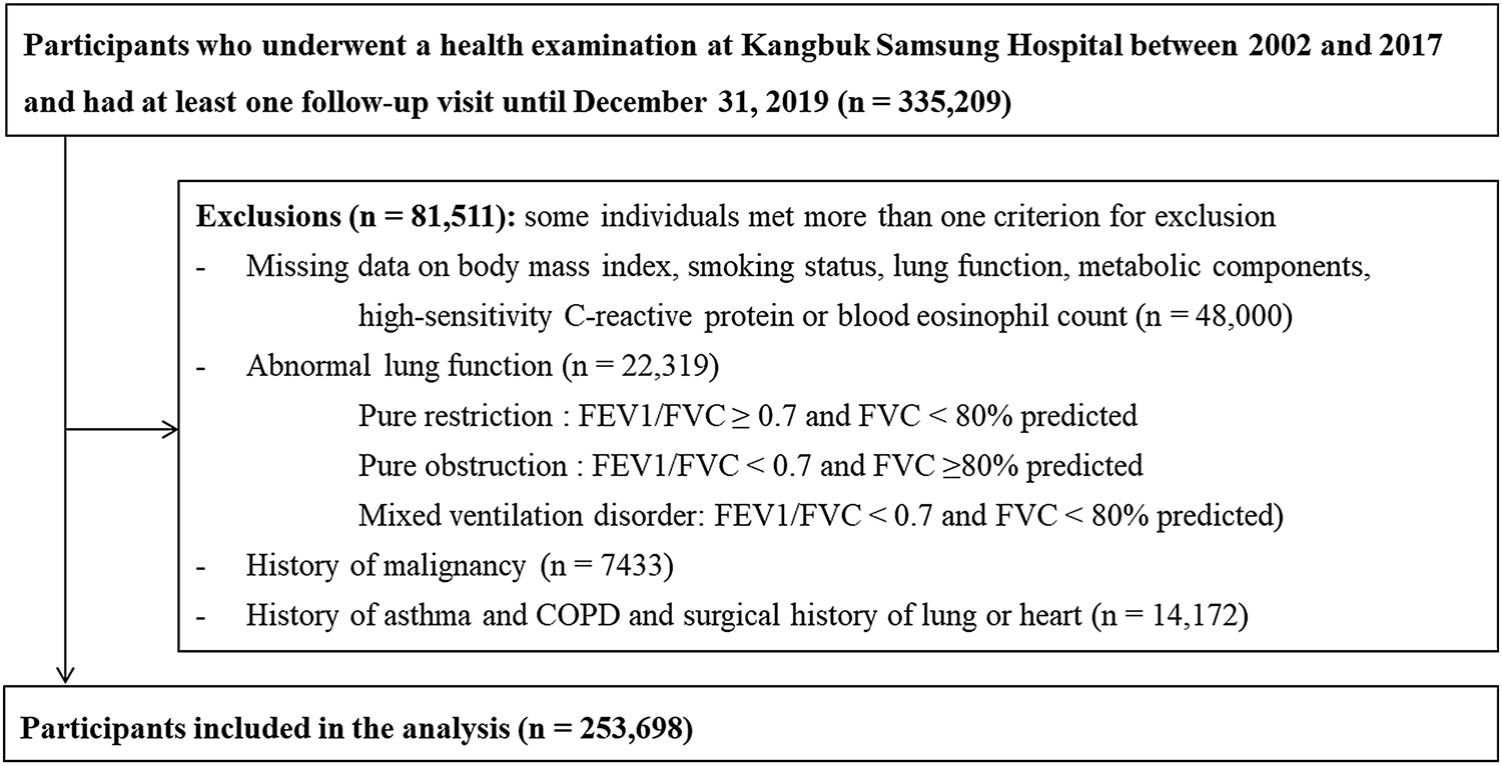
Flow chart for selecting the study population. Out of the 81,511 participants who were excluded, 71,801 met only one exclusion criterion, 9027 met two exclusion criteria, 663 met three exclusion criteria, and 20 participants met four exclusion criteria.

The present cohort study included participants with at least one follow-up visit between January 1, 2011, and December 31, 2019 (n = 335,209). After the exclusion of 81,511 participants, 253,698 participants were ultimately included in the analysis (Fig. 1).

This study was approved by the Institutional Review Board of Kangbuk Samsung Hospital (KBSMC 2022-03-055), which waived the requirement for written informed consent owing to the use of de-identified data obtained as part of routine health screening examinations. All procedures involved in this study of human participants were in accordance with the ethical standards of the institutional research committee and with the 1964 Helsinki declaration and its later amendments or comparable ethical standards.

### Data collection

At baseline and follow-up visits, data on demographic characteristics, medical history, medication use, smoking status, physical activity level^17^, usual dietary intake, and other lifestyle habits were collected via standardised, self-administered questionnaires. Smoking status was categorised as never, former, or current smoker. Average alcohol consumption was calculated based on the frequency and amount consumed per drinking day and then categorised as none or < 20 and ≥ 20 g ethanol/day. Physical activity levels were classified as inactive, minimally active, and health-enhancing physical activity (HEPA)^17^. Dietary intake was assessed using a validated 106-item food frequency questionnaire, with portion sizes and consumption frequency recorded. Nutrient values were calculated using a Korean food composition table^18^.

Body height and weight were measured by trained nurses, with participants wearing a hospital gown and no shoes. BMI was classified according to Asian-specific criteria^19^: underweight, < 18.5 kg/m^2^; normal weight, 18.5–23 kg/m^2^; overweight, 23–25 kg/m^2^; and obese, ≥ 25 kg/m^2^. Hypertension was defined as systolic blood pressure (BP) ≥ 140 mmHg, diastolic BP ≥ 90 mmHg, or current use of antihypertensive medication.

Blood samples were obtained after participants had fasted for at least 10 h. Fasting blood measurements included glucose, glycated haemoglobin, lipid, insulin, and hsCRP levels. Insulin resistance was assessed using the following homeostasis model assessment of insulin resistance (HOMA-IR) equation: fasting blood insulin (µU/mL) × fasting blood glucose (mmol/L)/22.5. Diabetes was defined as fasting serum glucose ≥ 126 mg/dL, glycated haemoglobin ≥ 6.5%, or current insulin or antidiabetic medication use.

Metabolically healthy (MH) persons were defined as having none of the following metabolic abnormalities, as previously applied^20^: (1) fasting glucose level ≥ 100 mg/dL or current glucose-lowering agent use, (2) BP ≥ 130/85 mmHg or current BP-lowering agent use, (3) elevated triglyceride level (≥ 150 mg/dL) or current lipid-lowering agent use, (4) low high-density lipoprotein cholesterol (HDL-C) (< 40 mg/dL in men or < 50 mg/dL in women), or (5) insulin resistance, defined as HOMA-IR score ≥ 2.5. In contrast, metabolically unhealthy (MU) was defined as having one or more of these metabolic abnormalities.

Spirometry was performed according to American Thoracic Society and European Respiratory Society recommendations^21^, using the Vmax22 system (Sensor-Medics, Yorba Linda, CA, USA)^16^. Forced expiratory volume in 1 s (FEV_1_) and forced vital capacity (FVC) were obtained in a pre-bronchodilatory setting. The predicted FEV1 and FVC values were calculated using equations for a representative Korean population sample^22^. To calculate the predicted FVC% and predicted FEV1%, we divided the measured value (L) by the predicted value (L) and converted the quotient into a percentage. The FEV1 to FVC ratio (FEV1/FVC) was calculated, and the actual measurements were used. Spirometry-defined lung function was classified as the restrictive pattern (RP, FEV_1_/FVC ≥ 0.7 and FVC < 80% predicted) and obstructive pattern (OP, FEV_1_/FVC < 0.7)^21^.

### Statistical analyses

Baseline characteristics of the participants were summarised by BMI category. The primary endpoints were RP and OP development. To assess for linear trends, we used the number of BMI categories as a continuous variable and tested it in each model. The follow-up time was calculated from the baseline examination to lung disease development or to the last health examination, whichever occurred first. Because the exact time of lung disease onset was unknown and occurred between the visit for lung disease diagnosis and the previous visit, we used flexible parametric proportional hazard models to account for this type of interval censoring^23^. This survival model parameterised log cumulative hazards as natural cubic splines of log time with three internal knots at the 25th, 50th, and 75th percentiles, respectively^23,24^. We estimated the hazard ratios (HRs) and 95% confidence intervals (CIs) for incident lung disease comparing BMI category to the normal BMI category as the reference, overall and separately for MH and MU individuals. Interactions by metabolic health status were examined using likelihood ratio tests comparing models with and without multiplicative interaction terms.

The models were first age- and sex-adjusted and then further adjusted for other potential confounding factors including study centre (Seoul or Suwon), examination year (1-year categories), smoking status (never, former, or current), alcohol consumption (none, < 20 g/day, ≥ 20 g/day, or unknown), physical activity level (inactive, minimally active, HEPA, or unknown), education level (high school graduate or less, college graduate or higher, or unknown), and total calorie intake (in quintiles or missing). We also fitted additional models adjusted for potential intermediate variables, including blood eosinophil count and hsCRP level, to evaluate potential mediators of the association between BMI and incident lung disease. Finally, to evaluate the effects of BMI changes and other covariates over time during follow-up, we conducted additional analyses with BMI and confounders as time-varying covariates in the models. The proportional hazards assumption was assessed by examining graphs of estimated log (–log) survival; ultimately, no violation of this assumption was found. We assessed collinearity among covariates using the variance inflation factor (VIF), and found no evidence of collinearity, with all VIF values less than 10. To conduct linear trend analyses, we included the median BMI values for each BMI category as continuous variables with linear terms in the regression models. To assess a quadratic trend, we centered the linear trend variable at the reference (normal-weight category) and then squared it.

To assess the longitudinal associations between the obesity category and subsequent changes in FEV1 and FVC over time, we used linear mixed models using random intercepts and slopes, while adjusting for potential baseline confounders. We estimated the annual change in FEV1 (95% CIs) from baseline, as well as the mean difference in annual FEV1 change comparing each BMI subgroup category with the reference group (normal-weight group). The same analyses were repeated for FVC.

All analyses were performed using Stata version 16 (StataCorp LP; College Station, TX, USA). *P* values < 0.05 were considered statistically significant.

## Results

At baseline, the mean (standard deviation) age of the participants was 37.4 ± 7.7 years, and 57.3% were male (Table 1; Fig. 1). The prevalence of underweight, normal weight, overweight, and obesity were 5.3%, 43.6%, 22.5%, and 28.7%, respectively, and the prevalence of MUH individuals with at least one metabolic syndrome component or insulin resistance was 46.4%. Increased BMI was positively associated with age; male sex; alcohol consumption; current smoking status; physical activity level; BP; fasting glucose, total cholesterol, triglyceride, LDL-C, HOMA-IR, and hsCRP levels; and eosinophil count, whereas BMI was inversely associated with HDL-C. Regarding spirometry values, increasing BMI categories were positively associated with FEV1 (L) and FVC (L), whereas FEV1/FVC decreased with increasing BMI categories.

**Table 1:** Baseline characteristics of study participants by body mass index category.

Characteristic	Overall	BMI category (kg/m^2^)				*p* for trend
		< 18.5	18.5–22.9	23.0–24.9	≥ 25	
Number	253,698	13,329	110,587	57,048	72,734	
Age (years)^a^	37.4 (7.7)	33.9 (5.9)	36.5 (7.4)	38.6 (8.1)	38.5 (7.8)	< 0.001
Sex (%)	57.3	9.9	38.0	74.2	81.8	< 0.001
Current smoker (%)	21.7	6.4	14.2	26.3	32.1	< 0.001
Alcohol intake (%)^b^	23.8	7.4	15.8	28.3	35.0	< 0.001
HEPA (%)^c^	15.7	9.1	14.5	17.5	17.5	< 0.001
Higher education (%)^d^	84.3	84.0	84.3	85.1	83.8	0.159
Energy intake (kcal/d)^e,f^	1521 (1154–1925)	1358 (999–1733)	1448 (1085–1836)	1569 (1210–1969)	1632 (1262–2065)	< 0.001
Metabolic parameters						
Hypertension (%)	9.6	1.2	4.1	10.2	19.0	< 0.001
Diabetes (%)	3.1	0.4	1.3	3.3	6.2	< 0.001
Systolic BP (mmHg)^a^	109.2 (12.9)	98.9 (9.6)	104.3 (11.4)	111.5 (11.5)	116.8 (12.1)	< 0.001
Diastolic BP (mmHg)^a^	69.9 (9.8)	64.1 (7.6)	66.8 (8.7)	71.3 (9.3)	74.7 (9.9)	< 0.001
Glucose (mg/dl)^a^	94.6 (13.8)	88.7 (7.9)	91.7 (10.8)	95.7 (13.6)	99.1 (17.1)	< 0.001
Total cholesterol (mg/dl)^a^	193.2 (34.0)	178.2 (28.5)	186.3 (31.5)	196.9 (33.7)	203.5 (35.4)	< 0.001
LDL-C (mg/dl)^a^	120.1 (31.9)	98.8 (24.3)	111.3 (29.0)	125.8 (30.9)	132.9 (32.0)	< 0.001
HDL-C (mg/dl)^a^	58.8 (15.3)	71.5 (14.6)	64.3 (14.9)	55.8 (13.4)	50.4 (12.1)	< 0.001
Triglycerides (mg/dl)^e^	90 (63–134)	62 (50–78)	73 (56–100)	100 (72–143)	130 (92–185)	< 0.001
HOMA-IR^e^	1.21 (0.80–1.80)	0.83 (0.56–1.20)	0.99 (0.67–1.40)	1.25 (0.87–1.77)	1.79 (1.23–2.60)	< 0.001
Inflammatory parameters						
hsCRP (mg/L)^e,g^	0.40 (0.20–0.90)	0.20 (0.20–0.40)	0.30 (0.20–0.60)	0.50 (0.30–0.90)	0.80 (0.40–1.50)	< 0.001
Eosinophil count^a^	2.1 (1.2–3.4)	1.8 (1.0–3.0)	1.9 (1.1–3.2)	2.2 (1.3–3.6)	2.3 (1.4–3.6)	< 0.001
Pulmonary function test						
FEV_1_ (L)^a^	3.41 (0.68)	2.91 (0.46)	3.23 (0.65)	3.58 (0.66)	3.64 (0.63)	< 0.001
FEV_1_ (% pred)^a^	99.4 (10.0)	98.1 (9.5)	100.0 (10.1)	99.5 (9.9)	98.6 (9.9)	< 0.001
FVC (L)^a^	4.05 (0.84)	3.28 (0.52)	3.78 (0.79)	4.32 (0.80)	4.40 (0.77)	< 0.001
FVC (% pred)^a^	98.1 (9.8)	94.9 (8.9)	98.3 (9.8)	98.7 (9.7)	97.9 (9.9)	< 0.001
FEV_1_/FVC^a^	84.5 (5.9)	88.9 (6.1)	85.7 (6.2)	83.1 (5.4)	82.7 (4.8)	< 0.001

Data are presented as ^a^means (standard deviations), ^e^medians (interquartile ranges), or percentages.

^b^ ≥ 20 g of ethanol per day; ^c^ HEPA was defined as meeting either of the following criteria: (1) vigorous-intensity activity on three or more days per week accumulating ≥ 1,500 metabolic equivalent of task (MET) min/week or (2) seven days of any combination of walking, moderate-intensity activities, or vigorous-intensity activities, achieving at least 3000 MET min/week; ^d^ ≥ college graduate; ^f^among 180,985 participants with plausible estimated energy intake levels (within three standard deviations from the log-transformed mean energy intake); ^g^among 123,194 participants without missing hsCRP values.

*BMI* body mass index, *BP* blood pressure, *FEV*_*1*_ forced expiratory volume in 1 s, *FVC* forced vital capacity, *HDL-C* high-density lipoprotein cholesterol, *HEPA* health-enhancing physical activity, *HOMA-IR* homeostasis model assessment of insulin resistance, *hsCRP* high-sensitivity C-reactive protein, *HOMA-IR* homeostasis model assessment of insulin resistance, *LDL-C* low-density lipoprotein cholesterol, *pred* predicted.

Table 2 presents the association of the BMI category with the overall incidence of RP in MH and MU participants. The median follow-up period was 4.9 years (interquartile range, 2.8–6.9; maximum, 8.8 years). The median frequency of follow-up visits, excluding the baseline visit, was 4 visits (interquartile range: 2–6 visits). During 1,221,414 person-years of follow-up, 10,775 new-onset cases of RP were identified, with an incidence rate of 8.8 cases per 10^3^ person-years. The associations between BMI category and incident RP were reverse J-shaped (*P* for quadratic trend < 0.001) and obesity was associated with an increased risk of RP in MH and MU groups but this association was more evident in MU than in MH individuals (*P* for interaction = 0.001). After adjustment for age, sex, other confounders (model 1), the multivariable-adjusted HRs (95% CIs) for incident RP comparing underweight, overweight, and obesity with normal weight were 1.90 (1.74–2.08), 0.91 (0.83–0.98), and 1.15 (1.05–1.25), respectively, in the MH group, and 1.97 (1.65–2.35), 1.07 (1.00–1.15), and 1.38 (1.30–1.47), respectively, in the MU group. When changes in BMI and other confounders during follow-up were updated as time-varying covariates, similar associations were observed. Further adjustment for hsCRP level and blood eosinophil count did not qualitatively change the association between BMI category and RP.

**Table 2:** Development of restrictive lung diseases by body mass index category in metabolically healthy and unhealthy phenotypes.

BMI category (kg/m^2^)	Person-years	Incident cases	Incidence rate (cases per 10^3^ PY)	Age- and sex-adjusted HR (95% CI)	Multivariable-adjusted HR^a^ (95% CI)		HR (95% CI)^b^ (in the model using time-dependent variables)
					Model 1	Model 2	
Total (n = 270,190)							
< 18.5	62,646.0	732	11.7	1.89 (1.75–2.05)	1.90 (1.75–2.05)	1.90 (1.75–2.06)	2.17 (2.00–2.35)
18.5–22.9	531,375.3	3969	7.5	1.00 (reference)	1.00 (reference)	1.00 (reference)	1.00 (reference)
23.0–24.9	277,652.6	2307	8.3	1.03 (0.97–1.08)	1.03 (0.97–1.08)	1.02 (0.97–1.08)	0.94 (0.89–0.99)
≥ 25.0	349,740.1	3767	10.8	1.39 (1.32–1.46)	1.38 (1.32–1.45)	1.37 (1.31–1.44)	1.34 (1.28–1.41)
*P* for linear trend				< 0.001	< 0.001	< 0.001	< 0.001
*P* for quadratic trend				< 0.001	< 0.001	< 0.001	< 0.001
Metabolically healthy (n = 128,548)							
< 18.5	54,008.0	597	11.1	1.89 (1.73–2.07)	1.90 (1.74–2.08)	1.90 (1.74–2.08)	2.19 (2.00–2.40)
18.5–22.9	370,170.1	2426	6.6	1.00 (reference)	1.00 (reference)	1.00 (reference)	1.00 (reference)
23.0–24.9	128,735.9	771	6.0	0.91 (0.84–0.99)	0.91 (0.83–0.98)	0.90 (0.83–0.98)	0.83 (0.76–0.90)
≥ 25.0	92,426.4	674	7.3	1.16 (1.06–1.27)	1.15 (1.05–1.25)	1.14 (1.04–1.24)	1.11 (1.02–1.20)
*P* for linear trend				< 0.001	< 0.001	< 0.001	< 0.001
*P* for quadratic trend				< 0.001	< 0.001	< 0.001	< 0.001
Metabolically unhealthy (n = 141,642)							
< 18.5	8638.0	135	15.6	1.96 (1.65–2.34)	1.97 (1.65–2.35)	1.98 (1.66–2.36)	2.12 (1.77–2.54)
18.5–22.9	161,205.2	1543	9.6	1.00 (reference)	1.00 (reference)	1.00 (reference)	1.00 (reference)
23.0–24.9	148,916.8	1536	10.3	1.07 (1.00–1.15)	1.07 (1.00–1.15)	1.07 (1.00–1.15)	1.00 (0.93–1.07)
≥ 25.0	257,313.7	3093	12.0	1.40 (1.32–1.49)	1.38 (1.30–1.47)	1.37 (1.29–1.46)	1.37 (1.28–1.46)
*P* for liner trend				< 0.001	< 0.001	< 0.001	< 0.001
*P* for quadratic trend				< 0.001	< 0.001	< 0.001	< 0.001

*P* = 0.001 for the overall interaction between metabolic health status and BMI category for incident restrictive lung diseases (adjusted model 1).

^a^Estimated from parametric proportional hazard models. Multivariable model 1 was adjusted for age, sex, centre, year of screening examination, education level, smoking status, alcohol intake, physical activity level, and total energy intake; model 2: model 1 plus adjustment for eosinophil count and high-sensitivity C-reactive protein level.

^b^Estimated from parametric proportional hazard models with BMI category, smoking status, alcohol intake, physical activity level, and total energy intake as time-dependent categorical variables and baseline age, sex, centre, year of screening examination, and education level as time-fixed variables.

*BMI* body mass index, *CI* confidence interval, *HR* hazard ratio, *PY* person years.

Table 3 presents the association of BMI with the overall incidence of OP in MH and MU participants. During 1,226,550 person-years of follow-up, 7140 new-onset cases of OP lung disease were identified, with an incidence rate of 5.8 cases per 10^3^ person-years. The BMI category was inversely associated with incident OP lung disease, and these associations were more pronounced in MU than in MH individuals (*P* for interaction = 0.001). The multivariable-adjusted HRs (95% CIs) for OP risk comparing underweight, overweight, and obesity with normal weight were 0.95 (0.80–1.12), 0.88 (0.81–0.96), and 0.72 (0.65–0.80), respectively, among MH individuals and 1.28 (0.96–1.71), 0.87 (0.80–0.94), and 0.59 (0.55–0.64), respectively, among MU individuals. These associations between BMI category and OP were similarly observed in time-dependent analyses and in analyses with further adjustments for hsCRP level and blood eosinophil count. Sensitivity analyses that excluded BMI outliers did not alter the results (Table S1). Additionally, the results were not qualitatively affected in sensitivity analyses that included average alcohol consumption and dietary intake as continuous variables (Table S2). In analyses using linear mixed models, we also examined the association between obesity category and serial change in absolute FVC and FEV1 values (Table S3). The annual decline in both FVC and FEV1 values was greater in the overweight and obesity categories compared with the normal weight category. Compared to FEV1, the mean difference in annual change was higher for FVC in the overweight and obesity categories compared to the normal-weight category.

**Table 3:** Development of obstructive lung diseases by body mass index category in metabolically healthy and unhealthy phenotypes.

BMI category (kg/m^2^)	Person-years	Incident cases	Incidence rate (cases per 10^3^ PY)	Age and sex-adjusted HR (95% CI)	Multivariable-adjusted HR^a^ (95% CI)		HR (95% CI)^b^ (in the model using time-dependent variables)
					Model 1	Model 2	
Total (n = 270,190)							
< 18.5	63,901.9	200	3.1	1.02 (0.89–1.18)	1.01 (0.88–1.17)	1.01 (0.87–1.17)	0.99 (0.85–1.16)
18.5–22.9	533,215.9	2911	5.5	1.00 (reference)	1.00 (reference)	1.00 (reference)	1.00 (reference)
23.0–24.9	277,246.6	2056	7.4	0.88 (0.83–0.94)	0.87 (0.82–0.92)	0.87 (0.82–0.93)	0.89 (0.84–0.95)
≥ 25.0	352,185.6	1973	5.6	0.64 (0.60–0.68)	0.62 (0.58–0.66)	0.62 (0.58–0.65)	0.61 (0.58–0.65)
*P* for linear trend				< 0.001	< 0.001	< 0.001	< 0.001
*P* for quadratic trend				< 0.001	< 0.001	< 0.001	< 0.001
Metabolically healthy (*n* = 128,548)							
< 18.5	55,017.4	152	2.8	0.96 (0.81–1.14)	0.95 (0.80–1.12)	0.94 (0.80–1.12)	0.92 (0.77–1.11)
18.5–22.9	371,738.5	1687	4.5	1.00 (reference)	1.00 (reference)	1.00 (reference)	1.00 (reference)
23.0–24.9	128,470.8	771	6.0	0.89 (0.81–0.97)	0.88 (0.81–0.96)	0.88 (0.81–0.96)	0.91 (0.84–1.00)
≥ 25.0	92,550.4	479	5.2	0.73 (0.66–0.81)	0.72 (0.65–0.80)	0.72 (0.65–0.80)	0.69 (0.62–0.76)
*P* for linear trend				< 0.001	< 0.001	< 0.001	< 0.001
*P* for quadratic trend				< 0.001	< 0.001	< 0.001	< 0.001
Metabolically unhealthy (n = 141,642)							
< 18.5	8884.5	48	5.4	1.29 (0.97–1.73)	1.28 (0.96–1.71)	1.28 (0.96–1.71)	1.24 (0.91–1.69)
18.5–22.9	161,477.4	1224	7.6	1.00 (reference)	1.00 (reference)	1.00 (reference)	1.00 (reference)
23.0–24.9	148,775.8	1285	8.6	0.88 (0.82–0.95)	0.87 (0.80–0.94)	0.87 (0.80–0.94)	0.89 (0.82–0.96)
≥ 25.0	259,635.2	1494	5.8	0.62 (0.57–0.67)	0.59 (0.55–0.64)	0.59 (0.55–0.64)	0.59 (0.55–0.64)
*P* for linear trend				< 0.001	< 0.001	< 0.001	< 0.001
*P* for quadratic trend				< 0.001	< 0.001	< 0.001	< 0.001

*P* = 0.001 for the overall interaction between metabolic health status and BMI category for incident obstructive lung diseases (adjusted model 1).

^a^Estimated from parametric proportional hazard models. Multivariable model 1 was adjusted for age, sex, centre, year of screening examination, education level, smoking status, alcohol intake, physical activity level, and total energy intake; model 2: model 1 plus adjustment for eosinophil count and high-sensitivity C-reactive protein level.

^b^Estimated from parametric proportional hazard models with BMI category, smoking status, alcohol intake, physical activity level, and total energy intake as time-dependent categorical variables and baseline age, sex, centre, year of screening examination, and education level as time-fixed variables.

*BMI* body mass index, *CI* confidence interval, *HR* hazard ratio, *PY* person years.

Finally, we evaluated the effect of metabolic health status on lung function impairment within the same BMI category (Table 4). The MH group was considered the reference for each BMI stratum. The incident RP risk significantly increased in MU participants across all BMI categories compared to that in the MH group. Conversely, the incident OP risk did not significantly differ between the MH and MU groups in normal-weight and overweight individuals, although the incident OP risk was significantly lower in the MU compared with the MH group in the obese stratum (adjusted HR = 0.81, 95% CI 0.73–0.89).

**Table 4:** Development of restrictive lung diseases by metabolically healthy status stratified by body mass index category.

BMI category (kg/m^2^)	Person-years	Incident cases	Incidence rate (per 10^3^ PY)	Age and sex-adjusted HR (95% CI)	Multivariable-adjusted HR (95% CI)^a^	HR (95% CI)^b^ (in time-dependent model)
Restrictive lung disease						
Normal weight						
Metabolically healthy	370,170.1	2426	6.6	1.00 (reference)	1.00 (reference)	1.00 (reference)
Metabolically unhealthy	161,205.2	1543	9.6	1.11 (1.04–1.19)	1.14 (1.07–1.22)	1.16 (1.08–1.24)
Overweight						
Metabolically healthy	128,735.9	771	6.0	1.00 (reference)	1.00 (reference)	1.00 (reference)
Metabolically unhealthy	148,916.8	1536	10.3	1.31 (1.20–1.43)	1.35 (1.24–1.47)	1.39 (1.26–1.52)
Obesity						
Metabolically healthy	92,426.4	674	7.3	1.00 (reference)	1.00 (reference)	1.00 (reference)
Metabolically unhealthy	257,313.7	3093	12.0	1.34 (1.24–1.46)	1.38 (1.27–1.50)	1.49 (1.36–1.62)
Obstructive lung disease						
Normal weight						
Metabolically healthy	371,738.5	1687	4.5	1.00 (reference)	1.00 (reference)	1.00 (reference)
Metabolically unhealthy	161,477.4	1224	7.6	1.00 (0.93–1.08)	0.98 (0.91–1.06)	1.00 (0.92–1.08)
Overweight						
Metabolically healthy	128,470.8	771	6.0	1.00 (reference)	1.00 (reference)	1.00 (reference)
Metabolically unhealthy	148,775.8	1285	8.6	1.00 (0.91–1.09)	0.96 (0.88–1.05)	0.92 (0.84–1.00)
Obesity						
Metabolically healthy	92,550.4	479	5.2	1.00 (reference)	1.00 (reference)	1.00 (reference)
Metabolically unhealthy	259,635.2	1494	5.8	0.84 (0.76–0.94)	0.81 (0.73–0.89)	0.81 (0.73–0.90)

^a^Estimated from parametric proportional hazard models. The multivariable model was adjusted for age, sex, centre, year of screening examination, education level, smoking status, alcohol intake, physical activity level, and total energy intake.

^b^Estimated from parametric proportional hazard models with BMI category, smoking status, alcohol intake, physical activity level, and total energy intake as time-dependent categorical variables and baseline age, sex, centre, year of screening examination, and education level as time-fixed variables.

*BMI* body mass index, *CI* confidence interval, *HR* hazard ratio, *PY* person years.

## Discussion

In the current cohort study, both obesity and metabolic health status were independently associated with increased RP risk. Participants with obesity showed a higher incidence of RP in both MH and MU groups, although this association was stronger in the MU group. Likewise, a significantly increased RP risk was observed in MU individuals, across all BMI categories. Conversely, OP risk decreased as BMI increased in both MH and MU groups. Although both FVC and FEV1 declined more annually in obese participants when compared to normal-weight participants, the mean difference in annual decline was higher for FVC than for FEV1. Consequently, the inverse association between obesity and OP risk observed in our study was attributed to the greater decline in FVC among obese individuals, rather than FEV1. To our knowledge, this is the first longitudinal study demonstrating the differential impact of BMI and metabolic health status on lung function impairment risk, depending on spirometry parameters.

Previous reports also showed the harmful effects of obesity^6,10,12^ and metabolic abnormalities^1,8,9,14,25^ on RP. Obesity mechanically causes RP by decreasing the diaphragm and compromising chest wall compliance, resulting in limited lung expansion and decreased lung volume. Moreover, the most conceivable factor for the association between RP and metabolic abnormalities is insulin resistance, which is significantly higher in patients with RP than in patients with OP; therefore, the major effect of insulin resistance may be on lung tissue, with a slight effect on airway diameter^14,26^. Insulin resistance reduces glucose utilisation and induces abnormal fat metabolism in skeletal muscles, possibly impairing mitochondrial ATP production and reducing skeletal muscle strength^27^. As forced respiration during spirometry requires respiratory skeletal muscle contraction, insulin resistance may mediate a decline in lung function, especially a greater decline in FVC than FEV1^28^. Insulin resistance-related hyperglycaemia^29^ can also cause non-enzymatic glycosylation of collagen and elastin in the lung and chest wall, leading to consequent stiffening of the thorax and lung parenchyma^30^; increased RP risk may be closely associated with a combination of the mechanical effect of obesity and metabolic effect of insulin resistance. Accordingly, in our study, the association between obesity and RP risk was stronger in MU than in MH individuals.

Interestingly, in contrast to a previous cross-sectional study^31,32^, we demonstrated that MHO was not harmless, especially for RP. In addition to temporal ambiguity owing to the cross-sectional design, this study defined MHO as having two or fewer metabolic syndrome components. Because impaired lung function risk is related to each metabolic parameter^1,14,25,33^ and the number of metabolic components^33,34^, a less strict definition for MH may not have provided a clear comparison of obesity per se with normal weight MH individuals. Overweight and underweight individuals were included in the reference group. This definition of comparison groups makes the findings difficult to interpret because of the harmful effect of being underweight on lung function^35^, which was also observed in our study. The longitudinal nature of our study, the strict definition of MH, choice of MH normal-weight participants as the reference group, and availability of repeated BMI measurements and metabolic health status and incorporation in the analysis possibly allowed us to reveal the effects of MHO on lung function.

In our study, obesity and metabolic abnormalities were associated with decreased OP risk, especially in the obese stratum. Until now, the effects of obesity and metabolic syndrome on OP have been controversial, varying from a negative^25,36^ to positive association^1,11,12^, although generally no association has been observed concerning metabolic syndrome or its components^1,9,14,37^. The reasons for the mixed results and inverse association between obesity, metabolic unhealthiness, and OP risk in our cohort are unclear. Previous studies have shown an association of OP with systemic inflammation^38^, not metabolic syndrome^9,14,26^. However, systemic inflammation is largely dependent on the degree of obesity, especially abdominal obesity and obesity commonly coexists with metabolic abnormalities and systemic inflammation^39^; thus, it is difficult to evaluate the effect of obesity versus other accompanying metabolic and inflammatory factors given their interrelationship. In our study using linear mixed models, a greater decline in both FVC and FEV1 was observed in obese than normal-weight participants, despite the inverse association between obesity and OP risk based on FEV1/FVC ratio. Thus, a more pronounced decline in FVC than FEV1 by obesity mechanisms may be an explanation because this change can result in a higher FEV1/FVC ratio^36^, leading to positive correlations between obesity and FEV1/FVC ratios. Furthermore, the impact of obesity on OP may be underestimated when diagnosing OP with conventional screening spirometry, which is performed to measure lung function based on patient effort, including deep breaths and forced expiration. This measurement likely obliterates the impact of obesity, particularly on FEV1. In addition, functional airway debility may go undetected in screening spirometry for healthy subjects, because FEV1/FVC < 0.7 predominantly reflects large airway obstruction^40^. Therefore, careful consideration is required when assessing OP based on screening spirometry, especially in healthy young and middle-aged subjects.

In the current study, both obesity and metabolic abnormalities appeared to be important risk factors for RP, even in apparently healthy individuals, although their effect on OP appears to be complicated to determine based on conventional spirometry. Our findings have several important clinical implications. RP is associated with increased mortality and cardio-metabolic diseases^3,41^. Therefore, this study has important strengths because it demonstrates the potential role of modifiable obesity and metabolic health status on impaired lung function, given the projected growing public health impact of lung function^3,6,41^ and the high prevalence of obesity and metabolic syndrome^8,12^.

However, our study has several limitations. First, our results were obtained from young and middle-aged asymptomatic and relatively healthy Korean adults who participated in a regular health check-up program. Therefore, our findings cannot be generalised to other demographic populations. Second, BMI was used as a measure of obesity. However, its inability to distinguish between the composition and distribution of fat and muscle mass could cause individuals with similar BMI to have very different body compositions and metabolic profiles. Finally, we did not examine the long-term effects of obesity or metabolic abnormalities on lung function. Because an increased risk of adverse clinical outcomes by metabolic abnormalities may occur only after 8–10 years^42^, the follow-up duration (median 4.7 years) of the current study might have been relatively short to evaluate the apparent effect of obesity and metabolic abnormalities on lung function.

In conclusion, both BMI and metabolic health status appear to affect lung dysfunction, and their association varies depending on lung disease type. Obesity, even MHO, is not a harmless condition, especially for RP. Our results suggest that an RP of lung function impairment may be regarded as a pulmonary manifestation of metabolic syndrome and obesity. Therefore, maintaining a healthy weight and remaining MH may help prevent chronic lung diseases. However, the pathophysiological mechanisms involved in different relationships according to lung function impairment patterns require further study.

## Supplementary Information

Supplementary Information.

## Data Availability

The data are not publicly available outside of the hospital because of Institutional Review Board restrictions (the data were not collected in a way that could be distributed widely). However, the analytical methods are available from the corresponding author upon request.
