# Supplementary material for: Metabolically healthy and unhealthy obesity and the development of lung dysfunction
Source: Sci Rep. 2023 Mar 27;13:4938. doi: 10.1038/s41598-023-31960-7 (PMC10042802; doi:10.1038/s41598-023-31960-7)
Supplement: Supplementary file 1 — Supplementary Information. [file 41598_2023_31960_MOESM1_ESM.docx]

| **Table S1.** Development of restrictive lung diseases / obstructive lung diseases by body mass index category in metabolically healthy and unhealthy phenotypes after excluding 3,339 subjects with BMI outliers | | |
| --- | --- | --- |
| BMI category (kg/m^2^) | Multivariable-adjusted HR^a^ (95% CI) for restrictive lung diseases | Multivariable-adjusted HR^a^ (95% CI) for obstructive lung diseases |
|  |  |  |
| Total subjects |  |  |
| <18.5 | 1.90 (1.75-2.05) | 1.01 (0.87-1.17) |
| 18.5–22.9 | 1.00 (reference) | 1.00 (reference) |
| 23.0–24.9 | 1.03 (0.97-1.08) | 0.87 (0.82-0.93) |
| ≥25.0 | 1.35 (1.29-1.42) | 0.62 (0.58-0.66) |
| *P* for linear trend | <0.001 | <0.001 |
| *P* for quadratic trend | <0.001 | <0.001 |
| Metabolically healthy |  |  |
| <18.5 | 1.90 (1.74-2.08) | 0.95 (0.80-1.12) |
| 18.5–22.9 | 1.00 (reference) | 1.00 (reference) |
| 23.0–24.9 | 0.91 (0.83-0.98) | 0.89 (0.81-0.97) |
| ≥25.0 | 1.12 (1.02-1.22) | 0.73 (0.65-0.8) |
| *P* for linear trend | <0.001 | <0.001 |
| *P* for quadratic trend | <0.001 | <0.001 |
| Metabolically unhealthy |  |  |
| <18.5 | 1.96 (1.64-2.34) | 1.28 (0.96-1.70) |
| 18.5–22.9 | 1.00 (reference) | 1.00 (reference) |
| 23.0–24.9 | 1.07 (1.00-1.15) | 0.87 (0.80-0.94) |
| ≥25.0 | 1.36 (1.27-1.45) | 0.59 (0.55-0.64) |
| *P* for linear trend | <0.001 | <0.001 |
| *P* for quadratic trend | <0.001 | <0.001 |
| *P for interaction* | <0.001 | 0.002 |

^a^Estimated from parametric proportional hazard models.

BMI outliers were defined as 1.5 times of the interquartile range below the first quartile or above the third quartile of data, which corresponded to BMIs less than 14.2 or greater than 32.1 kg/m^2^.

Multivariable model 1 was adjusted for age, sex, centre, year of screening examination, education level, smoking status, alcohol intake, physical activity level, and total energy intake.

Abbreviations: BMI, body mass index; CI , confidence interval; HR, hazard ratios

| \| **Table S2.** Development of restrictive lung diseases / obstructive lung diseases by body mass index category in metabolically healthy and unhealthy phenotypes among 242,225 ssubject without missing information of both alcohol intake and total energy intake \| \| \| \| --- \| --- \| --- \| \| BMI category (kg/m^2^) \| Multivariable-adjusted HR^a^ (95% CI) for restrictive lung diseases \| Multivariable-adjusted HR^a^ (95% CI) for obstructive lung diseases \| \| \| Total subjects \|  \|  \| \| <18.5 \| 1.90 (1.75-2.07) \| 1.00 (0.86-1.16) \| \| 18.5–22.9 \| 1.00 (reference) \| 1.00 (reference) \| \| 23.0–24.9 \| 1.01 (0.96-1.07) \| 0.87 (0.82-0.93) \| \| ≥25.0 \| 1.38 (1.31-1.45) \| 0.62 (0.58-0.66) \| \| *P* for linear trend \| <0.001 \| <0.001 \| \| *P* for quadratic trend \| <0.001 \| <0.001 \| \| Metabolically healthy \|  \|  \| \| <18.5 \| 1.93 (1.76-2.13) \| 0.94 (0.79-1.12) \| \| 18.5–22.9 \| 1.00 (reference) \| 1.00 (reference) \| \| 23.0–24.9 \| 0.90 (0.82-0.98) \| 0.89 (0.81-0.97) \| \| ≥25.0 \| 1.15 (1.05-1.26) \| 0.72 (0.65-0.81) \| \| *P* for linear trend \| <0.001 \| <0.001 \| \| *P* for quadratic trend \| <0.001 \| <0.001 \| \| Metabolically unhealthy \|  \|  \| \| <18.5 \| 1.87 (1.55-2.26) \| 1.25 (0.92-1.69) \| \| 18.5–22.9 \| 1.00 (reference) \| 1.00 (reference) \| \| 23.0–24.9 \| 1.06 (0.98-1.14) \| 0.87 (0.8-0.94) \| \| ≥25.0 \| 1.37 (1.28-1.46) \| 0.60 (0.55-0.65) \| \| *P* for linear trend \| <0.001 \| <0.001 \| \| *P* for quadratic trend \| <0.001 \| <0.001 \| \| *P for interaction* \| 0.002 \| 0.005 \| |
| --- | --- | --- | --- | --- | --- | --- | --- | --- | --- | --- | --- | --- | --- | --- | --- | --- | --- | --- | --- | --- | --- | --- | --- | --- | --- | --- | --- | --- | --- | --- | --- | --- | --- | --- | --- | --- | --- | --- | --- | --- | --- | --- | --- | --- | --- | --- | --- | --- | --- | --- | --- | --- | --- | --- | --- | --- | --- | --- | --- | --- | --- | --- | --- | --- | --- | --- | --- | --- | --- | --- | --- | --- |

^a^Estimated from parametric proportional hazard models. Multivariable model 1 was adjusted for age, sex, centre, year of screening examination, education level, smoking status, alcohol intake (continuous variable), physical activity level, and total energy intake (continuous variable).

Abbreviations: BMI, body mass index; CI , confidence interval; HR, hazard ratios

**Table S3.** Annual changes in absolute fev1 and fvc by body mass index category in metabolically healthy and unhealthy phenotypes

|  | FEV1 | | FVC | |
| --- | --- | --- | --- | --- |
|  | Annual change  in absolute value | Multivariable-adjusted  average difference in annual change | Annual change  in absolute value | Multivariable-adjusted  average difference in annual change |
| Total participants |  |  |  |  |
| <18.5 | ▼0.019 (▼0.020–▼0.018) | 0.008 (0.007–0.009) | 0.010 (0.009–0.010) | 0.011 (0.011–0.012) |
| 18.5–22.9 | ▼0.027 (▼0.027–▼0.027) | 0.00 (reference) | ▼0.002 (▼0.002–▼0.001) | 0.00 (reference) |
| 23.0–24.9 | ▼0.032 (▼0.032–▼0.032) | ▼0.004 (▼0.005–▼0.004) | ▼0.015 (▼0.015–▼0.015) | ▼0.013 (▼0.013–▼0.013) |
| ≥25.0 | ▼0.029 (▼0.030–▼0.029) | ▼0.002 (▼0.002–▼0.002) | ▼0.020 (▼0.020–▼0.019) | ▼0.018 (▼0.018–▼0.017) |
| P for linear trend | <0.001 | <0.001 | <0.001 | <0.001 |
| P for quadratic trend | <0.001 | <0.001 | <0.001 | <0.001 |
| Metabolically healthy phenotype |  |  |  |  |
| <18.5 | ▼0.018 (▼0.019–▼0.017) | 0.007 (0.007–0.008) | 0.010 (0.010–0.011) | 0.009 (0.008–0.010) |
| 18.5–22.9 | ▼0.025 (▼0.026–▼0.026) | 0.00 (reference) | 0.001 (0.001–0.001) | 0.00 (reference) |
| 23.0–24.9 | ▼0.031 (▼0.032–▼0.031) | ▼0.005 (▼0.006–▼0.005) | ▼0.012 (▼0.012–▼0.011) | ▼0.013 (▼0.013–▼0.012) |
| ≥25.0 | ▼0.031 (▼0.032–▼0.031) | ▼0.005 (▼0.006–▼0.005) | ▼0.020 (▼0.020–▼0.019) | ▼0.020 (▼0.021–▼0.020) |
| P for linear trend | <0.001 | <0.001 | <0.001 | <0.001 |
| P for quadratic trend | <0.001 | <0.001 | <0.001 | <0.001 |
| Metabolically unhealthy phenotype |  |  |  |  |
| <18.5 | ▼0.024 (▼0.026–▼0.023) | 0.006 (0.005–0.007) | 0.007 (0.005–0.008) | 0.014 (0.012–0.016) |
| 18.5–22.9 | ▼0.030 (▼0.031–▼0.030) | 0.00 (reference) | ▼0.007 (▼0.008–▼0.007) | 0.00 (reference) |
| 23.0–24.9 | ▼0.032 (▼0.032–▼0.032) | ▼0.002 (▼0.002–▼0.001) | ▼0.017 (▼0.018–▼0.017) | ▼0.010 (▼0.011–▼0.009) |
| ≥25.0 | ▼0.029 (▼0.029–▼0.029) | 0.001 (0.001–0.002) | ▼0.020 (▼0.020–▼0.019) | ▼0.012 (▼0.013–▼0.012) |
| P for linear trend | <0.001 | <0.001 | <0.001 | <0.001 |
| P for quadratic trend | <0.001 | <0.001 | <0.001 | <0.001 |

Estimated from linear mixed models with random intercepts and slopes, with FEV1 as the continuous outcome.

The multivariable model was adjusted for smoking status, alcohol consumption, physical activity level, and total energy intake as time-dependent categorical variables and baseline age, sex, centre, year of screening examination, and education level as time-fixed variables.

Abbreviations: BMI, body mass index; CI , confidence interval; FEV_1_, forced expiratory volume in 1s; FVC, forced vital capacity; ▼, negative
